# Language impairment in progressive supranuclear palsy and corticobasal syndrome

**DOI:** 10.1007/s00415-019-09463-1

**Published:** 2019-07-18

**Authors:** Katie A. Peterson, Karalyn Patterson, James B. Rowe

**Affiliations:** Department of Clinical Neurosciences and MRC Cognition and Brain Sciences Unit, University of Cambridge, Herchel Smith Building for Brain and Mind Sciences, Forvie Site, Robinson Way, Cambridge, CB2 0SZ UK

**Keywords:** Corticobasal syndrome, Language, Progressive supranuclear palsy

## Abstract

Although commonly known as movement disorders, progressive supranuclear palsy (PSP) and corticobasal syndrome (CBS) may present with changes in speech and language alongside or even before motor symptoms. The differential diagnosis of these two disorders can be challenging, especially in the early stages. Here we review their impact on speech and language. We discuss the neurobiological and clinical-phenomenological overlap of PSP and CBS with each other, and with other disorders including non-fluent agrammatic primary progressive aphasia and primary progressive apraxia of speech. Because language impairment is often an early and persistent problem in CBS and PSP, there is a need for improved methods for language screening in primary and secondary care, and more detailed language assessments in tertiary healthcare settings. Improved language assessment may aid differential diagnosis as well as inform clinical management decisions.

## Introduction

Language impairment is increasingly recognised as a feature of many neurodegenerative conditions and is not restricted to the primary progressive aphasias (PPA). In this review, we focus on the speech and language changes caused by progressive supranuclear palsy (PSP) and the corticobasal syndrome (CBS), and their relationship to PPA. We aim to show how the assessment and understanding of language deficits can aid the diagnosis of PSP and CBS.

Different regions within the brain’s language network are susceptible to different neuropathologies, leading to language syndromic profiles which provide clues to the underlying pathological process. This is most evident in the group of disorders known collectively as PPA. For example, tau-positive pathologies with frontal perisylvian degeneration are associated with the non-fluent/agrammatic variant of PPA (nfvPPA; previously termed progressive non-fluent aphasia, PNFA), and also with apraxia of speech (AOS) [1–3]. In contrast, the semantic variant (svPPA) is strongly associated with tau-negative TDP43-positive pathology and marked anterior temporal atrophy [1, 4, 5], while Alzheimer’s disease (AD) pathology is the principal cause of the logopenic variant of PPA (lvPPA) [6]. Such associations may also hold for the language impairments arising from PSP and CBS.

PSP and CBS are both complex, clinically heterogeneous disorders, sometimes described as “Parkinson plus” syndromes or “atypical parkinsonism”. In the original 1964 case series of nine PSP patients [7], cognitive changes were described in seven cases, and were often prominent. However, the disease became defined by its akinetic rigidity and oculomotor paresis as primarily a movement disorder. PSP was also considered a prototypical “subcortical dementia” with cognitive slowing, distinct from the cortical dementias [8], although the cognitive profile of PSP is highly suggestive of additional cortical atrophy and pathology [9]. The 2017 revised clinical diagnostic criteria for PSP redress the balance, with cognitive subtypes of PSP supported by neuropathological validation, including “PSP-SL” with speech and language impairments [10, 11].

CBS has had a similar nosological journey, with “early dementia” as an exclusion in early diagnostic criteria [12]. More recently, cognitive changes, including language impairment, have been shown to be prominent in CBS as well as PSP [13–16] and may be the presenting symptoms in around half of cases [13, 17–19]. The current clinical diagnostic criteria for CBS [20] provide for cognitive subtypes, including a non-fluent/agrammatic variant of CBS (CBS-NAV). Here, we review the literature to characterise the linguistic profiles associated with PSP and CBS and suggest simple tools for health care professionals to use in assessment of language in suspected cases of both conditions.

It is important to note that most of the evidence to date comes from ‘classic’ phenotypes and often with clinical diagnosis in the absence of pathological verification. One must also distinguish between the clinical *syndromes* (*PSPs* and *CBS*) and histopathologically confirmed cases which we refer to as *PSPd* and corticobasal degeneration, *CBD*. As emphasised by Mesulam et al. [21], two additional factors interfere with straightforward classifications and summaries of evidence: (1) degenerative conditions often change their character during disease progression, either bringing new features into the picture or changing the prominence of existing symptoms; (2) some patients manifest patterns of features that do not match the syndromes outlined in consensus clinical diagnostic criteria.

## Clinical and brain imaging features

### Progressive supranuclear palsy

PSPs was first described by Steele, Richardson, and Olszewski in 1964 as a progressive neurodegenerative disease characterised by vertical supranuclear opthalmoplegia, pseudobulbar palsy, dysarthria, dystonic rigidity of the neck and upper trunk, and *dementia* [7]. Neuropathological investigation indicated nerve cell loss, neurofibrillary tangles, gliosis, and demyelination in the basal ganglia, brain stem, and cerebellum. PSPd is classified as a primary tauopathy [22, 23]. MRI investigations show midbrain and frontal atrophy, while parietal and temporal regions are relatively spared [24].

Since its first description, a number of clinical variants of PSP have been defined based on clinical presentation, severity, and neuropathological findings. The term ‘Richardson’s syndrome’ (PSP-RS) or Steele–Richardson–Olszewski syndrome now refers to the classically described clinical presentation. The clinical criteria for PSP proposed by the National Institute of Neurological Disorders and Stroke and the Society for PSP are highly predictive of PSP pathology, i.e., PSPd [25]. However, in a multicentre study of 100 autopsy-confirmed cases of PSPd, only 24% had originally presented as PSP-RS [18], although many others develop PSP-RS features with time. New criteria for PSPs have since been developed by the Movement Disorder Society-endorsed PSP study group (MDS-PSP), to accommodate the various clinical presentations associated with PSPs [10]. The 2017 MDS-PSP criteria identify speech and language as a core functional domain, and operationalise the diagnosis of the PSP-SL speech/language phenotype (see Table 1 for a description).

**Table 1:** The language features of PSP-SL, CBS-NAV, and nfvPPA

PSP-SL [10]	CBS-NAV [20]	nfvPPA [35]
At least one of the following features, which has to be persistent (rather than transient): (1) non-fluent/ agrammatic variant of primary progressive aphasia (loss of grammar and/or telegraphic speech or writing), or (2) progressive apraxia of speech (effortful, halting speech with inconsistent speech sound errors and distortions or slow syllabically segmented prosodic speech patterns)	Effortful, agrammatic speech plus at least one of: (1) impaired grammar/sentence comprehension with relatively preserved single-word comprehension, or (2) groping, distorted speech production (apraxia of speech)	At least one of: (1) agrammatism in language production; and (2) effortful, halting speech with inconsistent speech sound errors and distortions (apraxia of speech). PLUS at least two of: (1) impaired comprehension of syntactically complex sentences (2) spared single-word comprehension (3) spared object knowledge

*PSP-SL* PSP with predominant speech/language disorder, *CBS-NAV* CBS presenting as nfvPPA, *nfvPPA* non-fluent/agrammatic variant of primary progressive aphasia

### Corticobasal syndrome

The clinical syndrome of CBS is defined by the combination of motor deficits (typically progressive asymmetrical akinetic rigidity, dystonia, tremor, myoclonus) and deficits in higher cortical functions (alien limb, apraxia, cortical sensory change) [26, 27]. This syndrome was associated with the specific tau-pathology called corticobasal degeneration (CBD, or formerly cortico-basal-ganglionic-degeneration). However, pathologically-proven CBD has been associated with a range of clinical syndromes including behavioural variant frontotemporal dementia (bvFTD), progressive AOS, PPA, and PSPs [28, 29]. Likewise, the syndrome of CBS is associated with pathologies other than CBD including PSPd, frontotemporal lobar degeneration with ubiquitin- and TDP-43-positive inclusions (FTLD-TDP), AD, Pick’s disease, Lewy body disease, as well as mutations in the microtubule-associated protein tau and progranulin genes [30, 31].

On neuroimaging, CBS typically reveals asymmetric cortical atrophy, particularly in frontal and parietal regions, and atrophy of the basal ganglia. However, patterns of atrophy may vary according to the underlying pathological process and phenotype [32]. In some cases, CBD and PSPd have both been associated with greater atrophy of the prefrontal, premotor or supplemental motor regions [32], compared to temporal regions [28, 33]. These patients tended to present with a behavioural or language syndrome partially resembling bvFTD, nfvPPA, or with executive dysfunction. Where atrophy is marked in temporoparietal regions in CBS, the underlying pathology may be AD [32, 33], while prominent frontotemporal atrophy is suggestive of FTLD-TDP pathology [32].

Cognitive changes, including impairment in language, memory, executive functions, visuospatial abilities and social cognition are common in patients with CBS and are often the first presenting symptoms [13, 16, 17, 20, 34]. Armstrong and colleagues [20] operationalised a non-fluent agrammatic clinical phenotype of CBS (CBS-NAV; see Table 1). As Table 1 demonstrates, there is substantial overlap across the language features of PSP-SL, CBS-NAV, and nfvPPA as described in their respective diagnostic criteria.

## Speech

Dysarthria is common in PSPs [18, 36–38], including a mixed dysarthrophonia with hypokinetic, spastic and, in some cases, ataxic components [37–40]. The features of the dysarthria are consistent with the observed neurodegeneration of the midbrain, globus pallidus, striatum, and hypothalamic nucleus [38]. Abnormal characteristics of speech in PSPs can include reduced articulatory velocity, prolonged speech pauses, inappropriate silences, deficits in vowel articulation, harsh voice, stuttering, echolalia, palilalia, and hypophonia [37, 38, 40–47].

Dysarthria is also noted in patients with CBS [17, 48–51] and is characterised by a disturbance of the temporal and prosodic aspects of speech [49]. In comparing CBS and Parkinson’s disease (PD), while both groups show dysprosodic features such as monopitch and monoloudness, PD patients have predominant vocal abnormalities (e.g., harsh or breathy voice) whereas CBS patients are more likely to show abnormalities in temporal organisation (e.g., slow rate, dysfluency, and prolonged duration of phonemes) [49]. Features suggested as indicative of CBD pathology include orobuccal apraxia [17, 52, 53] and flat aprosodic speech [15].

## Language features

Language impairment was once thought to be uncommon in PSPs/PSPd and CBS/CBD [8, 54], but some early studies may have overlooked or downplayed language disturbances to emphasise the importance of motor features [55]. Additionally, formal language testing was often not included, or described, in early studies. More recent studies suggest that language impairment is common in CBS [16, 17, 51, 56] and PSPs [57–59], which we summarise here.

### Fluency

PSPs and CBS typically result in severely impaired initial letter fluency [9, 60–77] and moderate deficits in category fluency [9, 41, 60, 64, 67–69, 74, 76–78]. The fact that letter fluency is typically more affected than category fluency [9, 64] is consistent with the finding, in functional imaging with healthy adults, that letter fluency principally activates frontal regions whereas category fluency produces mainly temporal-lobe activation [79, 80].

Additionally, the types of words generated are revealing: PSPs and CBS patients sometimes, puzzlingly, generate low-frequency words [81]. For example, *ptarmigan, pterodactyl*, and *farinaceous* were the only p- and f-words elicited from a PSP-RS patient in our clinic. This is distinct from the strong bias towards high-frequency words in svPPA and AD [82].

#### Executive vs. language accounts of fluency impairment

Fluency scores may relate to other measures of language ability [83], but it is commonly accepted that fluency performance depends upon additional cognitive processes including executive function, initiation, working memory and attention [84]. Therefore, without more detailed language assessment, it would be unclear whether a fluency deficit reflects a breakdown of language per se, other cognitive processes, or both.

Schofield et al. reported impaired letter fluency in 100% of 11 autopsy-confirmed PSP-RS patients (PSPd), while 55% were impaired on a composite score derived from various language tests including category fluency [75]. Similarly, confrontation naming was dramatically better than letter fluency in a PSPs study by Gerstenecker et al. [85]. This suggests that, in at least a subset of patients, fluency impairment in PSP is independent from problems in the principal aspects of language, although of course language deficits, when present, may exacerbate the fluency impairment. Indeed, Sitek et al. [86] reported language impairment (based on assessment of spontaneous speech, naming, comprehension, and repetition) in 80% of 20 patients with PSP-RS and even hypothesised that this might underlie the low Frontal Assessment Battery scores observed in PSPs.

Patients with PSPs and CBS have reduced initiation and speed of processing which might in part explain their poor fluency, though this would likely have a similar impact on letter and category fluency. In a study of over 300 PSPs cases [62], the most salient cognitive impairment was on the ‘initiation and perseveration’ subscale of the Dementia Rating Scale, although this is a composite score derived from tasks which include verbal fluency. While the slowed speed of processing in PSPs and CBS patients [57, 73, 87] could contribute to their fluency impairment (since these tasks are timed), extending the time limit beyond one minute rarely improves the number of words elicited in letter fluency from people with PSPs: they appear to dry-up after a few words regardless of the time available. Consistent with this are findings from Robinson and colleagues [88] showing that the majority of words generated over a range of fluency tasks by a PSPs patient with frontal dynamic aphasia were produced within the first 20–50% of the allotted time.

#### Neural correlates of fluency impairment

Investigations of neuroanatomical correlates of fluency impairment in PSPs seem to implicate the dorsolateral prefrontal cortex (DLPFC), concordant with both lesion [89] and functional imaging studies [90] associating letter fluency with DLPFC, especially on the left side. Verbal fluency (combining letter and category fluency) in PSPs has been shown to correlate with a component of the informant-rated Katz Adjustment Scale which is purported to correspond to DLPFC functioning [91]. Further, impaired letter fluency apparently relates to increased neuronal tau deposition in the superior frontal gyrus [75], an area associated with the executive aspects of the task [92], while a composite language score (including category fluency) was related to pathology in inferior frontal and perirhinal cortices, areas associated with word retrieval [92, 93]. These findings suggest that executive dysfunction underlies the letter fluency impairment in PSPs, though they do not definitively establish whether executive function is vital in fluency tasks generally or is differentially critical to the initial-letter version.

Neural correlates of fluency impairment in CBS have not been extensively investigated. Positron emission tomography indicates left frontoparietal hypoperfusion in CBS patients with impaired verbal fluency [94], suggesting the presence of a broader dysexecutive syndrome. In pathologically-confirmed CBD [19], histopathologic abnormalities were most prominent in frontal and parietal regions in patients who also showed neuropsychological impairment, again consistent with an account involving executive dysfunction.

### Naming

Confrontational naming appears to be relatively well preserved in PSPs. A mild naming impairment has been reported when an extended naming task is used such as the full 60-item version of the Boston Naming Test (BNT) [45, 67, 78, 85, 95, 96], while some studies using shortened naming tasks have found performance to be normal [69, 97] or only mildly impaired [57, 59]. This may suggest that the problem relates more to sustained attention than to the processes required for object naming per se.

Naming difficulty should not be confused with the more common description “word finding difficulty” during conversational speech, which is likely to reflect the fluency difficulties outlined above. Maher, Smith and Lees [71] reported mild word-finding difficulty in seven out of 25 PSPs patients without severe dysphasia or comprehension deficits. Nine of the 25 patients completed the Graded Naming Test [98], with all but one in the average or superior ranges. Impaired word-finding in connected speech was also reported by Kobylecki et al. [99] in 33% of 60 PSPs patients.

Where frank naming errors do occur in PSPs, they are often visually related to the target objects [36] and thus may be indirectly due to gaze palsy or other visual problems that are common in PSPs [36, 58, 100]. There are also a few reports of errors at the semantic or lexical retrieval stage [59, 95], though these could reflect attentional deficits rather than a genuine degradation of semantic knowledge.

Confrontational naming is impaired in some patients with CBS [17, 19, 56, 101, 102] but often only mildly so [101]. It has been suggested that this reflects impaired retrieval rather than a semantic deficit, since performance is usually aided by phonemic cueing [102, 103], but semantic errors have been reported [101]. Naming impairment in CBS has been shown to correlate with impaired lexical retrieval (assessed using a category fluency task), as well as with impaired visual-spatial functioning [102]. Further, in this study, naming impairment correlated with volume of the left lateral temporal cortex and the left frontal cortex, which, according to the authors’ hypotheses, contribute to lexical retrieval and verbal working memory, respectively.

The types of errors made during a naming task are rarely reported, leaving the basis for these errors in PSPs and/or CBS unclear. We suggest that future studies should analyse and report the types of naming errors in an attempt to specify the contribution of lexical, semantic, attentional, and visuospatial factors.

Some studies report an intriguingly disproportionate impairment for action naming in PSPs and CBS [104–106]. For example, in a study of frontotemporal dementia, PSPs, and CBS [104], the latter two groups had more difficulty with action than object naming which was attributed to disruption of frontoparietal-subcortical circuits involved in action knowledge and representation. Similarly, Chow et al. [105] found that PSPs and CBS patients were impaired on sound naming (naming the objects which produce various sounds), particularly for manipulable objects (defined as objects for which a goal-directed hand movement is required to produce the sound; e.g., guitar). Further, performance on this task was associated with atrophy of the left pre-motor region. This group was not significantly impaired on naming using the short form of the BNT (note that all but one of the items on this test are non-manipulable objects). These results might reflect the role of classical motor networks in speech production and language comprehension for actions [107].

### Comprehension and semantic association

Systematic investigation suggests that single-word comprehension is relatively spared in PSPs [100, 104, 108], while sentence comprehension may be mildly impaired [47, 57, 100, 109], reinforcing the impression in early studies [71, 110]. However, five out of six patients investigated by Podoll, Schwarz and Noth [36] and more than 50% of patients in a study by Catricalà et al. [59] showed impaired word as well as sentence comprehension. Comprehension of action-verbs may be particularly affected in PSPs, in keeping with the deficits in action naming above [106, 111, 112].

While semantic knowledge is relatively preserved in PSPs there can be a mild impairment on formal testing. For example, on the Sydney Language Battery (SYDBAT) PSPs patients were mildly impaired relative to controls, and not significantly different from patients with nfvPPA [57]. There are also reports of impaired performance on tests of semantic association [57, 59, 95], although there is a significant executive component to this test, and furthermore, scanning the test items requires vertical eye movements which may disadvantage patients with this disorder. Indeed, many assessments of semantic knowledge use word-picture or sentence-picture matching tasks on which deficits in visual attention or visual scanning may impact performance [100, 109].

In CBS, single-word comprehension was impaired in 52% of patients in Di Stefano et al. [17], but in only two of fifteen patients in Huang et al. [51], and no significant difference from controls was reported by Cotelli et al. [104]. However, differences between these studies in (a) the criteria for diagnosing CBS (b) the tests used to evaluate single-word comprehension (e.g., matching of names to real objects vs. pictures), and (c) the methods for classifying impairment (e.g., direct comparison to a group of control participants vs. normative values) make it difficult to draw generalised conclusions.

Patients with a clinical pattern of nfvPPA and pathologically proven CBD show significant impairment in sentence comprehension [113], particularly in later stages of the disease course [19]. The researchers argued that this deficit reflected genuinely impaired understanding of grammatically complex sentences rather than problems of single-word knowledge. Further detail was provided by Cotelli et al. [114] who reported unimpaired sentence comprehension in a CBS group as measured using a sentence-picture matching task, but impaired ability to detect violations of grammar during a sentence judgment task. The interpretation of a specific syntactic deficit may, however, be complicated by the fact that patients with CBS [101, 115] and pathologically proven CBD [19] can show impairments on tasks of semantic association. The difficulty in judging a sentence as ungrammatical might therefore be, in part, semantic.

### Sentence production

Agrammatism is uncommon in early PSP-RS, but features of nfvPPA or PSP-SL, including agrammatism, have been reported in cases of PSPd [21, 116]. Tasks requiring sentence production, such as picture description, can reveal other abnormalities, including: perseverations; fewer morphemes, words, and sentences; and fewer novel words and sentences in the context of normal syntactic structure [36, 58, 59, 100]. CBS, on the other hand, can present with syntactic errors, phonological errors, paraphasias, and speech apraxia [17, 51, 101] as well as impaired syntactic knowledge [114] and phonological processing [101]. Unfortunately, differences between these studies hinder clear and generalisable conclusions, including differences in diagnostic criteria, sample sizes, test materials and threshold values for impaired performance.

### Repetition

While repetition has not been extensively investigated in PSPs, one study reported that single-word repetition in this group was significantly worse than controls, but significantly better than a group with nfvPPA [57]. Four patients presenting with nfvPPA who later developed clinical features of PSP [117] were not significantly impaired on either single-word or sentence repetition relative to controls. Further, they performed significantly better on both repetition tasks than a group of patients with nfvPPA without PSP syndrome.

Deficits of word and sentence repetition have been noted in some CBS patients [17, 51, 53]. Impaired sentence repetition, which is characteristic of lvPPA, seems to be more common in CBS with underlying amyloid pathology [53].

### Reading

In PSPs, where tested, reading is described as slow with poor pronunciation due to dysarthria [36, 41, 117]. There are also reports of difficulties in deciphering words and/or in finding the beginning of each new line in a text, suggesting that visual scanning deficits are a significant factor in the reading difficulties in PSPs [36].

Patients with CBS may be unimpaired on single word reading [101], although a minority of patients make regularisation errors [51, 101] (e.g., pronouncing a written word like *sew* as “sue”, in line with more typical spelling-to-sound correspondences). One patient in a study by Graham et al. [101] who was severely aphasic made errors which were characterised as non-word approximations of the target (e.g., girl → “dirl”) or visual errors (e.g. loss → “lost”). The majority of CBS patients in this study were impaired on a task of non-word reading, consistent with their impaired performance on other tasks of phonological skills such as phoneme blending and segmentation. Larger studies with systematic analysis of reading in PSPs and CBS are so far lacking.

### Writing

Dysgraphia is common in PSPs and CBS [36, 51], and may be due to linguistic, cognitive, visual, or motor impairment. Writing features noted in PSPs include micrographia, abnormal slanting, omission of letters and words, addition of letters, and omission of diacritic marks in a Polish-language group [36, 59, 96, 118, 119]. The micrographia of PSPs differs from Parkinson’s disease by the absence of progressive fatigue (reducing letter size). The dysgraphia in PSPs may result from visuo-constructional or oculomotor deficits, whereby abnormal vertical and horizontal saccades disrupt the visual monitoring of writing [36, 118], and may be exacerbated by severe limb akinesia.

CBS and autopsy-proven CBD are associated with constructional apraxia and impaired handwriting, in keeping with the limb apraxia which affects most patients [19, 34]. Dysgraphia was claimed to be the most common language-related abnormality in CBS by Huang et al. [51], although its nature was not described and limb apraxia was noted in all patients. Nevertheless, errors both in writing and oral spelling (the latter used by Graham et al. [101] to avoid the impact of motor deficits on handwriting) in CBS have included article omissions, letter substitutions and omissions, and a mixture of phonologically plausible and non-phonologically plausible spelling errors [101, 120–122].

### Summary of language deficits

The language impairment in PSP seems largely consistent with a “frontal” deficit, possibly linked to executive dysfunction and impaired initiation. It has certainly been argued that executive deficits underlie the verbal fluency impairment in PSP [64, 123]. In contrast to the typically more severe deficit of category than letter fluency impairment in AD that suggests a breakdown of semantic memory [9, 64], the reverse is typical in PSP and could be explained by problems of initiation or general executive function. Indeed, patients with PSPs show impairments in other tests of ‘frontal functioning’ such as planning, orienting attention, and set-shifting [59, 67, 124–126]. Consistent with this are reports of an “adynamic aphasia” in PSP, characterised by reduced verbal output and cognitive processing speed [13, 57, 108]. In a recent study [57], the finding of only mild or borderline correlations between tests of language and those of executive function led the authors to conclude that language impairment in PSPs is not solely attributable to executive dysfunction. It is worth noting, however, that the PSPs cohort in this study were recruited from a predominantly cognitive disorders clinic and may therefore have presented with more cognitive and behavioural features than motor features. Future research is clearly needed to delineate the nature of the language impairment in PSP, including an analysis of the types of errors made by patients on language tasks to determine whether these can be “explained away” by visual or motor difficulties.

While visual and motor problems exacerbate some of the language impairments documented in CBS, these patients appear to experience a true breakdown of language ability [127]. Aphasic syndromes reported in CBS/CBD include a surprisingly wide variety: non-fluent aphasia, anomic aphasia, fluent aphasia, Broca’s aphasia, mixed aphasia, and AOS [17, 19, 28, 34, 55, 128]. These different patterns may be a function of the stage at which assessment occurs, or perhaps they will be found to relate to the different pathologies which may underlie CBS when these are better delineated.

Most of the evidence to date comes from studies of classic phenotypes. It is becoming clear from the few available larger clinicopathological series that phenotypic variation is common, including clinical overlap (e.g., PSP-CBS, and CBS-PSP) and non-classical phenotypes (e.g., PSP-SL, CBS-NAV) [11, 18, 20, 21, 28, 29], and more such studies are clearly needed.

In addition, most studies have been cross-sectional and therefore may not adequately characterise the onset and evolution of language deficits. Early vs. late language loss in PSPs and CBS may turn out to have important clinical and pathological implications. From the few longitudinal clinicopathologic studies, it seems that patients with PSPd who present initially with language impairment (PSP-SL) later develop typical motor features (such as falls, abnormal saccades or pursuit, and supranuclear gaze palsy); this distinction may help to distinguish PSP-SL from nfvPPA [11]. Similarly CBD patients presenting with nfvPPA subsequently often show extrapyramidal involvement before death [15]. Conversely, in CBS at least, patients presenting with typical movement disorders also often develop aphasia or behavioural change [129].

Given that language symptoms can vary by stage, different clinical tools may be required to elicit and characterise language disorders at different stages. For example, patients may write answers in early but not late stages; significant gaze palsy and visual disturbance in later stages of PSPs may limit the usefulness of language tests with visual stimuli; and the assessment of aphasia can be complicated by the development of dysarthrophonia.

## Clinical utility of language assessment for PSP and CBS

Speech and language assessment may have utility for the differential diagnosis of PSP and CBD, which clearly overlap clinically and pathologically with each other and with disorders such as AD and PD. Language assessment may also aid monitoring and prediction of prognosis. This section outlines the differential linguistic features of CBD and PSPd, and provides recommendations, based on our review, of clinical tools for the assessment of language in PSPs and CBS.

### Differential diagnosis

#### PSPd vs. CBD

The patterns of linguistic impairment in PSP and CBD clearly overlap. Clinicopathological case reports demonstrate that both PSPd and CBD can present with AOS and/or nfvPPA [128]. AOS is a motor speech disorder characterised by dysprosodic speech, distorted sound substitutions, phonological additions, repetitions and prolongations, and decreased articulatory accuracy [116, 128]. Originally described in association with post-stroke aetiology [130], AOS is increasingly recognised in neurodegenerative conditions, including a clinical syndrome of primary progressive AOS (PPAOS) [131]. PPAOS has been distinguished from nfvPPA, which is a language disorder defined by agrammatic speech/writing, telegraphic or truncated speech, and impaired syntactic comprehension [35]. To make matters more complicated, the clinical features of apraxia and aphasia often co-occur [128].

Many patients with AOS/nfvPPA go on to develop motor symptoms and/or have autopsy-confirmed PSP or CBD [132–135]. In other words, AOS or nfvPPA may be prodromes to PSPs or CBS. Whether a case is labelled PPA, PPAOS, PSPs or CBS may depend on the stage of disease at which a patient is referred and the diagnosis made, or the expertise and interest of the clinician in eliciting relevant features [128]. Nevertheless, there may turn out to be linguistic features which help to differentiate PSP from CBD pathology: while both AOS and nfvPPA occur in CBD, PSPd appears to be more commonly associated with AOS without nfvPPA [136]; meanwhile, patients who meet the MDS-PSP criteria for PSP-SL may be more likely to have CBD than PSPd [137].

#### PSP vs. PD

Idiopathic Parkinson’s disease is associated with mild impairment of speech production, including the domains of volume (hypophonia), fluency, grammaticality, naming, syntactic complexity, and discourse [138, 139]. However, there are discernible differences in linguistic features between PD and PSP.

Language impairment is apparent early in the disease course in some PSPs patients [66], whereas language or global cognitive impairment typically occurs late in PD [140]. Letter and category fluency are significantly more impaired in PSP relative to PD patients [59, 74, 77, 81, 96, 140]. Foley et al. [140] compared naming, comprehension, and spelling in the two disorders. The PSPs patients were significantly more impaired than the PD patients on one of two spelling tasks. There were no group differences in naming using the 30-item Graded Naming Test [98], but significantly impaired naming in PSPs compared to PD was reported in another study using the 60-item Boston Naming Test [96]. PSPs patients may have significant handwriting impairment relative to PD patients, consistent with their apraxia and vertical gaze palsy [96].

Catricalà et al. [59] conducted a more detailed investigation of linguistic features in Italian-speaking PSPs and PD patients. The PSPs group were significantly impaired relative to the PD group on a range of language tasks, including naming, single word and sentence comprehension, sentence repetition, reading, semantic association, and in the number of orthographic errors during writing, although the PD group were significantly older than the PSPs group. In comparing a subset of the PD patients who were matched in age to the PSPs patients, sentence repetition and single word comprehension were no longer significantly different between groups. The researchers also examined linguistic features using a connected speech task. Relative to controls, PSPs patients showed a lower speech rate, with fewer sentences, words, and information units but a greater number of pronouns, and poorer performance on two measures of discourse efficiency and effectiveness; PD patients were impaired only for discourse efficiency and effectiveness. In comparing PSPs and PD, PD patients produced fewer nouns and more incomplete sentences, while PSPs patients had a lower speech rate, and produced fewer words, sentences and information units. The researchers concluded that language impairment, particularly lexico-semantic and syntactic impairment, is more severe in PSPs than in PD.

Motor speech assessment may also help distinguish PSP from PD. PD is characterised by pure hypokinetic dysarthria with hypophonic, monotonous speech whereas PSPs produces more severe mixed dysarthrophonia [38, 41, 58]. Further, dysarthria typically is present earlier and progresses more rapidly in PSPs than in PD [58].

#### CBD vs. AD

CBS can be due to underlying CBD (CBS-CBD) but is also commonly caused by AD pathology (CBS-AD). Typical AD patients often show language impairment, particularly in category fluency, naming, semantic knowledge, and discourse-level processing, with relatively intact syntactic and phonological abilities [141]. The logopenic variant of PPA, which is associated with Alzheimer’s pathology, is characterised by particular difficulties with word retrieval and sentence repetition [35].

A more challenging problem is to distinguish CBS-CBD from CBS-AD. The former is often associated with atrophy of frontal regions, whereas cases of the latter more often show temporoparietal atrophy [32, 33]. Accordingly, CBS-CBD patients may present with frontal features such as nfvPPA [52]. Hu et al. [142] recorded AOS in four of eleven CBS-CBD patients but in none of five CBS-AD patients. This difference, though not statistically significant with small numbers, may provide a pointer to phenomena for future investigation. Unfortunately, detailed language assessment was not reported in this study; but the finding of fronto-temporal hypoperfusion in the CBS-CBD patients suggests that linguistic impairment might have been detected if assessed. CBS-AD, however, may show more severe sentence repetition impairment, consistent with findings localising damage to more posterior brain regions [17, 33, 53, 142]. Further evidence on the distinctions between CBS-CBD and CBS-AD will require larger series of pathologically proven cases, or the use of robust biomarkers of clinical pathology using cerebrospinal fluid analysis or amyloid imaging.

### Tools for the assessment of language in PSP and CBS

#### Brief bedside tests

Two brief bedside language tests which are especially useful for crude diagnostic classification of PSPs and CBS are verbal fluency and reading. Letter fluency is significantly more impaired in PSPs patients compared to several other neurodegenerative disorders including idiopathic PD [74, 81, 140], multiple system atrophy [74], and AD [64]. Indeed, the generation of seven or fewer words in 1 min of a letter fluency task accurately distinguishes PSPs from other movement disorders [81, 143]. Reading tasks may also have utility in diagnosis of PSPs and CBS: impaired reading of easy, regular words is consistent with PSP patients’ difficulties in deciphering words [36], while impaired non-word reading is consistent with the phonological difficulties seen in CBS [101].

#### Extended in-clinic assessment

Most of the speech and language tests used in PPA research, such as the Western Aphasia Battery [144] and the Boston Diagnostic Aphasia Examination [145], were designed for post-stroke aphasia and thus should be supplemented with language tests developed specifically for progressive aphasia, such as the Northwestern Anagram Test (NAT) and the Sydney Language Battery (SYDBAT). The NAT can be used to assess syntactic abilities in patients who have impaired speech production [146], so is suitable for patients with AOS or a non-fluent presentation of PSPs or CBS. The SYDBAT evaluates naming, word repetition, word comprehension, and semantic association [147].

There is a need for a short standardised evaluation of progressive aphasia, suitable to apply the clinical diagnostic criteria [148]. A recently developed language screening tool for use in neurodegenerative disorders is the Mini Linguistic State Examination (MLSE; www.mlsexam.com) designed to screen for language impairment and to classify different varieties of PPA. The MLSE contains subtests which span the most commonly affected linguistic components in PPA, and is based on the recommendations of current diagnostic guidelines [35]. Of note, the MLSE incorporates error type classification within its scoring system. The MLSE has been developed initially in English and Italian but has the potential to be adapted for other languages/cultures, which will enable comparison of PPA patterns across languages and should provide previously unavailable advances in our understanding of these conditions. Research centres interested in adapting the MLSE are advised to contact the study team via www.mlsexam.com. Note that at the time of press, the MLSE is undergoing validation testing in English and Italian, but will in due course be made freely available for clinical and academic research purposes.

Where it is available, more detailed neuropsychological assessment can be useful to detect subtle impairments, nuance subgrouping, and predict prognosis. It is important for a neurological/neuropsychological assessment to evaluate the impact of other factors on performance on language tests, including executive function, mood, memory, visual problems, and motor abilities. Where motor or visual problems are detected, assessment should be adapted appropriately, for example using tests which do not rely on visual processing (such as orally-presented syntactic comprehension tests in addition to a sentence-picture matching task). Presenting tests at eye-level may also facilitate assessment of PSP patients, who lack vertical eye movements [58].

The 2017 MDS-PSP criteria for clinical diagnosis of PSP will facilitate the characterisation of the neuropsychological and linguistic profile of the variants of PSPs [10]. For example, PSP-RS is associated with more severe cortical atrophy, particularly in frontal regions, than PSP-parkinsonism [149, 150] and this is hypothesised to underlie the more pronounced cognitive and executive dysfunction in PSP-RS. Verbal fluency was more impaired in PSP-RS and PSP-CBS than in PSP with predominant gait freezing (PSP-PGF) [151]. Prefrontal atrophy may be still more severe in PSPs with prominent nfvPPA [117]. PSP with predominant frontotemporal dysfunction is associated with the behavioural and cognitive changes typically seen in bvFTD, which have included reports of language impairment in up to 37% of patients [66]. PSPd presenting as CBS has been associated with progressive aphasia and specifically with nfvPPA [137, 152]. Additionally, micrographia is noted in patients with PSP-PGF [42, 43]. Such speech and language changes could be useful for predicting prognosis. For example, slowly progressing CBS patients were significantly more likely to have a motor speech disorder than rapid progressors, and there was a trend for more frequent dysgraphia [51].

## Conclusion

Although more research is needed to characterise the linguistic profiles of these conditions and their longitudinal patterns, there is already evidence to date that language assessment can play an important role in the clinical evaluation and management of patients with PSPs and CBS.
